# Selections in Pathology and Surgery; or, an Exposition of the Nature and Treatment of Local Disease, Exhibiting New Pathological Views, and Pointing out an Important Practical Improvement

**Published:** 1839-10

**Authors:** 


### Art. XV.

1. *Selections in Pathology and Surgery; or, an Exposition of the Nature and Treatment of Local Disease, exhibiting new pathological views, and pointing out an important practical improvement; illustrated by Cases.* By John Davies, Surgeon to the General Infirmary at Hertford.—*London,* 1839. 8vo, pp. 128.
2. *Practical Remarks on the use of Iodine locally applied in various Surgical Diseases and External Injuries; illustrated by Cases.* By John Davies, &c. &c.—*London,* 1839. 8vo, pp. 62.

The two small treatises which stand at the head of the present article constitute properly but one volume, the first mentioned containing the second. This last the author has published in a separate form, being desirous, he says, to make known the curative properties of iodine as extensively as possible amongst the members of the profession. We shall, in like manner, divide our remarks under two heads: first, considering the pathological views which are set forth; and secondly, the practical application of the remedy in question in various forms of disease.

Were we to be critical, we might fairly object to the title given to the first portion of this work, as conveying an idea of a variety of morbid states being therein discussed; whereas the whole is limited to an explanation of some of the phenomena of inflammation. In reviewing the more voluminous writings of Hunter and others, in our last two Numbers, we took occasion to enter so fully on this subject, in order to show the variety of opinions held respecting this most important of all diseases, that it will only be requisite for us to allude briefly to the views entertained by the author of the present pamphlet. Indeed, we do not hesitate to state our opinion, that no writings of the present description *can* enhance our knowledge of the pathology of inflammation; that we may go on reasoning and reasoning only, *ad infinitum,* without advancing one single step in the enquiry; and that, until some other physiologist steps into the field, prepared by his experimental researches to confirm what is known, or else to place the matter in an entirely new light, the subject, as far as words can determine it, may, for the present, be considered as exhausted. The microscope, we conceive, affords us the only means, at present, of investigating the true nature of inflammation. Nothing satisfactory can be elicited by reasoning unsupported by experiments; and next to nothing can be gained by considering the symptoms during life, or examining the alteration of structure left after death. Previous to carrying on, however, an investigation of this description, it is essential that the eye should be accustomed to watch the phenomena exhibited by the capillary vessels in a natural state, in order that we may commit as few mistakes as possible, in tracing and recording the changes presented to us by disease; and it is essential that our minds should not be tinctured with particular views respecting the structure and functions of the vessels concerned in the process; but that we follow up the plan, so successfully adopted by Kaltenbrunner, of simply detailing what is offered to the sense of sight, without reference to any peculiar theories.

Several reasons might be assigned for the difficulty we experience in arriving at an exact knowledge of the proximate cause of inflammation, besides the extreme minuteness of the parts concerned in the process: an important one is our ignorance concerning some of the leading functions of the vessels. On this ground have arisen the numerous controversies, carried on more particularly by those who have never made any experiments themselves, but who, picking and culling from the researches of others, determine, in their own minds and to their own satisfaction, what shall and what shall not be the actual condition of the vessels in an inflamed part. Aware of this defect, the author of the short treatise before us, prefaces his observations on the pathology of inflammation by some remarks on the mechanism, structure, and functions of the blood-vessels, in which he glances at a few of their leading properties.

"Notwithstanding all the theories," he says, "that have been advanced respecting the nature or proximate cause of inflammation, all must agree that the visible and tangible signs of it depend upon the condition of the arteries of the inflamed part and upon the modification of the circulation of the blood within them. Taking these facts as the groundwork of the enquiry, we shall proceed to examine what this condition really consists in; we may then be able to assign some rational or probable cause for the *inflammatory* appearance put on by so many diseases, possessing, in all other respects, such various properties, and leading to consequences so different in their nature. First, however, as our views differ essentially respecting the *natural functions* of the vessels principally engaged in the representation of the more striking phenomena of inflammation from those of any author with whose works we are acquainted, it is necessary to say a few words upon that subject." (p. 2.)

We must confess that we cannot detect anything either new or original in the views here put forth; and we think this will be obvious to our readers, on our laying before them an abridgment of Mr. Davies's statements, without any critical examination or special detail on our part.

The apparatus which conduces to carry on the circulation consists of a forcing machine, to which is attached an elastic tube, which tube divides and subdivides into innumerable branches. The heart is a muscular organ, its fibres being remarkably dense and with little cellular membrane intervening between them. Its contraction is *active*, but the dilatation of the cavities is *passive*, depending principally upon the *elasticity* of its structure. An explanation of the power by which "the muscular fibres of the heart are enabled to shorten themselves, below that medium which characterizes them as an elastic substance," although not "generally considered as pertaining to the *practical* part of the subject," the author very singularly conceives is "essential towards acquiring a rational idea of the pathology of inflammation." In the remarks which he deems it necessary to make on this point, and by which we are led to expect that the mystery will be cleared up, we are informed that organs and tissues, besides being composed of material molecules, have a *something* which confers upon them vitality, "that the properties and functions and effects of animate matter are only attributable to life, which an organ is capable of manifesting in a living state, and which it is not capable of exhibiting in the state of death," that for this particular reason the liver, "although as perfect for some time after death as before," refuses to secrete more bile, the stomach to digest more food, or the kidney to furnish any more urine; but that if we attempt to search into the *why* and the *wherefore* of these things, "our curiosity will be very likely to meet with disappointment." The arteries are considered as made up of three elastic tunics, the outermost, contrary to every other opinion and even ocular demonstration, being stated to possess elasticity in the least degree. No muscular fibres, after the minutest examination, can be detected in the middle coat. The vessels undergo no sensible motion during the circulation, and with the exception, in some instances, of a small portion of the aorta, are therefore mere passive tubes. They are always full of blood, each contraction of the left ventricle urging the column a step forward. If an artery be laid bare, and the finger placed very lightly upon it, no pulsation will be felt, while to the sight the vessel presents an unmoving cord. Two supposed prevalent errors are next combated, namely, that the arteries are always in a state of "forced distension," and pulsate independently of the heart. Having established these preliminary facts, respecting the condition of the heart and arteries during the natural and undisturbed circulation, the author next introduces us to that part of the subject upon which, we are told, mainly rest the new pathological views concerning inflammation. These are placed before the reader under two heads.

"1st. The only motion which the arteries undergo is that of *gradual* contraction and of *gradual* dilatation, so as to adapt themselves to the quantity of blood within them. 2d. The calibre of all the arterial branches during life and in a healthy state is below the medium of their elasticity. The overlooking or being ignorant of these two facts have led pathologists into endless absurdities respecting the pathology of inflammation; at any rate respecting the theory of the phenomena presented by it." (p. 10.)

We are then shown, in the ordinary way, that the arteries are always full of blood, whatever quantity may be in circulation; and that they have the faculty of accommodating themselves to their contents. While no mention is made in what part of the vessels this contractile power resides, and while every trace of muscularity is denied them, it is most infelicitously attempted to illustrate the operation of this power by reference to the action of parts, as the bladder or intestines, whose muscular structure is so very conspicuous:

"We now come," continues the author, "to the most important part of this subject. A material fact, which has been overlooked by physiologists, is that the caliber of the arterial tubes is always below that point which would obtain if the vessels were allowed to submit to their innate elastic forces. Instead of obeying the laws of dead matter, and of remaining at that state at which their elastic medium would place them, they are forced to submit to the vital force of contractility, and thereby reduce their canals to some extent below that medium." (p. 13.).

Do our readers require to be informed—what every physiological work proclaims, and every member of the profession knows—that the diameter of the arteries is greater after death than during life?—and yet this is the sum and substance, as far as we can see, of the "most important part of the subject," and the "material fact" which has been overlooked! Respecting the origin, distribution, physical or physiological properties of the capillary system, the immediate seat of disease, not a word is uttered.

We come, in the next place, to the "pathology of inflammation;" and here we regret being compelled to state at the outset that we have no favorable opinion to record; not that we quarrel with the author respecting the views he may choose to entertain, but that the matter introduced is, in many respects, so little connected with the subject under discussion, and the whole account of the process of inflammation, as it is seen going forward in the transparent textures, so miserably deficient, that the term "pathology," as here employed, is one almost of mockery. We shall extract those passages which show the condition of the vessels in an inflamed part, and contain all that is said respecting the nature of the disease. Alluding to the manner in which determinations of blood take place, it is said,

"Now, in the case of blushing, it is often that the face alone assumes increased redness. It may be asked, how do the arterial branches of one part of the body manage to acquire more blood than their due proportion? The heart pumps out the fluid equally for the benefit of all the branches. The power, therefore, of causing the disproportion must reside in the arteries themselves. Then comes the question—the most *important* question—by what process, or by what means, do the arteries of one seat succeed in obtaining more than their proportionate share of blood? The answer is short and clear—*simply and solely by enlarging their diameters or calibers in that seat.* . . . . Now, if the *cause* (meaning blushing) were different—if it were of such a nature as to weaken *permanently* the contractile powers of the vessels, more especially if it totally *destroyed* that power, *then* the vessels would be *incapable* of resuming their natural diameters; an undue proportion of blood would constantly exist in them; the seat of the disturbance would present the appearance of redness, and some degree of swelling, and furnish all the characters of incipient INFLAMMATION. In a word, the visible and tangible characters of inflammation depend entirely and solely upon an undue enlargement of the capillary extremities of the arteries. The enlargement may, and often does, extend some distance towards the larger branches, but its origin is invariably in the capillary tubes, and its extension takes place by continuity along the vessels. This enlargement enables them, as a matter of course, to hold more blood than the quantity naturally or proportionally belonging to them; which circumstance is the cause of the 'redness' of an inflamed part." (pp. 22-3.)

The variations in the degree of inflammation are shortly accounted for: "The cause may be of such a nature as merely to weaken the contractile force of the tubes in the smallest degree, or it may be such as totally to destroy that force. Between these two points there are various degrees, and, as a consequence, the inflammation may present various degrees of intensity."

From this it appears that Mr. Davies is to be classed with those who look upon inflammation as consisting only in relaxation and enlargement of the capillary vessels, and who thus manage to compress the whole philosophy of the disease into a nutshell. We do not intend to enter on this long-debated question; but we would remark, that such simple views, whether of health or disease, however ingenious, can seldom be just. They have their origin too frequently in the spirit of system, not in the careful study and enumeration of the complicated circumstances which concur in the production of all vital phenomena. How miserably do such views fail in explaining the series of morbid changes which constitute the entire process of inflammation. Let those who have never witnessed these various changes as they appear under the microscope to the eye, but peruse the writings of others who have made them the subject of their researches, and then they will become aware of the folly of attempting to reduce the intricate phenomena of the disease to a silly hypothesis concerning the "contraction or relaxation" of a few capillary tubes.

The abstract consideration of either theory certainly involves the doctrine of the vital contractility of the extreme branches—their independent power of altering the distribution of the blood, and of accelerating its motion. Unless we grant these, it will be impossible ever to illustrate the nature of inflammatory action. The author, however, with several others, has been led away in his attempt to resolve the local action of arteries into a relaxation of their tunics; and, did our space permit, we could adduce ample reason for rejecting this opinion. If we admit that these vessels are endowed with a vital contractility, by which their inner coat is always kept in close contract with the blood, then we must allow that, within certain healthy limits, they are also capable of distention; for the circulating fluid varies in quantity at different periods, and it is only when these limits are exceeded, either by loss of tonicity in the vessels, or by the pressure of a greater column of blood than they are able to sustain, that relaxation and debility ensue.

The next section is dedicated to an explanation of the "cause of difference in the character of inflammation." Our knowledge of the progress of this disease in different structures—since attention was first directed to it in this country by Dr. Carmichael Smyth, and afterwards in France by Pinel and Bichat—affords a beautiful illustration of the rich store of pathological treasures which may be accumulated by careful observation and research. Mr. Hunter was of opinion that the character of inflammation could not be determined by the tissue affected, otherwise we ought to expect various kinds of inflammation in an amputated limb. Even up to the present hour we have no evidence to show what is the difference, if there is any difference, in the primary phenomena which in one case leads to adhesion, in a second to suppuration, in a third to ulceration, and in a fourth to mortification. We say that the process may be the same, for all we know to the contrary, which leads to these various changes, requiring only, in some instances, to be increased or diminished, and, in others to meet with a structure which, from its peculiar organization or vital properties, is more ready to fall into one species of action than another. To say that "tissue modifies inflammation, or that inflammation is modified by tissue," is like reasoning in a circle; it is a substitution of words for knowledge—a cloak under which our ignorance may remain concealed. We are too well aware that no light will ever be shed on this point by mere conjecture; we are sick of the miserable theories which are invented; and, after all that has been done, we still think that a physiologist could not confer a greater boon on medical science, at this moment, than by giving a faithful relation of the phenomena of inflammation, as they arise in various tissues. How satisfactory would it be to be able to assign the just reason why the disease terminates in different ways under different circumstances; why it should be followed in one membrane by effusion of lymph, and in another by effusion of pus, instead of being compelled to receive these statements in the way they are now made. We do not despair of this task being yet accomplished; although, in order to trace out the steps which nature silently pursues, we should have many difficulties to contend with, many conflicting doubts to reconcile, and many anxious hopes to keep in abeyance.

The chapter in the work before us which has given rise to these reflections contains nothing on which the mind can rest. To us, indeed, many parts of it are incomprehensible, whilst the suggestions thrown out are such as we are neither prepared to receive nor reject. Could we bring ourselves to admit as unquestionable facts what the author adduces as such, and conscientiously believes to be so, we should be equally happy to rest in the conclusions he has drawn from them.

The *Selections in Pathology* conclude with a few remarks on the "condition of the blood" and the "influence of the nerves" in inflammation. While, in the former, there is little to commend, there is a good deal of what we conceive to be objectionable. In alluding to the morbid appearances observed in animals whose blood has been deprived of the greater part of its fibrine, it is attempted to show that the process of ulceration may be occasionally independent of inflammation. The illustrations adduced in proof are surely nothing less than absurd. They are—the *removal of structure upon a large scale, in cases of emaciation from want of food*—the fact, *that a stomach had, in one instance, consumed a portion of itself after death*—and the circumstance of *the ulceration which characterizes a chancre being often unattended by the least appearance of inflammation.* In speaking of the condition of the blood in inflammation the author seems inclined to the opinion that a morbid alteration in the circulating mass takes precedence of the local disorder. Instead, however, of addressing his observations to the question as it concerns inflammation, he enters upon the doctrine as it stands related to fever. With the latter we have nothing to do at present; but, as regards the former, we may be permitted to observe that, except where the blood contains an excess of fibrine, or is deficient in that material, the circulating mass seems to us to be *secondarily* affected.

The phenomena of the buffy coat, the causes of its formation, and the circumstances accompanying it, are scarcely touched upon, and what is advanced leaves the subject, to say the least, in no better condition than where it was found. That we may not be thought unjust, we shall give the following sentences without comment:

"Where the disposition to form the buff coat exists, if any number of portions of the same blood be drawn at the same time into different vessels of the same shape and size, the consistence of the cake will, in general, be found to vary in them all. The quantity of serum expressed, and the density of the coagulum, will be *least* in the portion drawn first, and both will *increase* in the order in which the different portions were abstracted. . . . . On the contrary, inflammatory blood will sometimes cease to be buffy after the first or second bleeding, though by far more commonly this quality *increases* in proportion to the quantity abstracted. It may be noticed, as a general rule, that the *thinner* and more *emaciated* the patient is, the denser and, generally, the quicker the coagulation will be. In *such constitutions* the blood presents the appearance of buff in the most *trifling* cases of inflammation; whereas, in *strong, robust,* especially fat, individuals, the fluid often exhibits hardly any buffiness, even in *severe* cases. The buff, as by far the more general rule, bears an *inverse* ratio to the vital powers of the system." (pp. 62-3.)

In concluding our remarks on the first part of this work, we must confess that we cannot understand what could have induced the author to give its contents to the world as exhibiting "new pathological views." Surely he cannot be ignorant of the fact, that all which he has advanced respecting the "functions of the blood-vessels," or the "pathology of inflammation," has been the subject of discussion for many years back; and we, for our parts, have not been able to discover any new light that has been thrown upon it.

We turn now with pleasure to the second part of the work, which consists of practical observations on the external use of iodine in various surgical cases. These observations are prefaced by some general remarks on local therapeutics, wherein the author takes the opportunity of stating his opinions respecting diseased action, the manner in which it takes place, and in which topical remedies succeed in removing it. Although we cannot coincide in the leading propositions laid down—and chiefly for a reason which may appear somewhat extraordinary to some, namely, that they are too simple to explain the intricate operations of nature—the chapter is, nevertheless, well worthy of a careful perusal. We will quote a passage which at once affords a key to the views brought forward, and will be found useful to remember as we proceed. After laying it down as the *grand principle* that disease is the same in its nature, whether situated externally or internally, and that during its progress secretion exceeds absorption, the author remarks:

"We have stated that when a part is undergoing disease, the secreting function generally overcomes the absorbent, which causes a preternatural deposit of matter. This is usually the first step towards the disorganization of the structure. The question to be now considered is, how does this condition of the part happen? It may, and probably generally does, owe itself to two causes: first, in consequence of the vital derangement of the capillary or secreting vessels, the calibers of these vessels enlarge, so as to enable more than the due proportion of fluid to pass through them; and, in the second place, as the absorbent function is the reverse of that of secretion, and must be performed by a *different* class of vessels, a similar derangement and relaxation must diminish their force, and thereby render their function *less* active than natural. For instance, if the vital derangement of the capillary extremity will cause this extremity, in consequence of its preternatural enlargement, to deposit *two* atoms in the time it could only deposit *one* in its natural state, it does not follow that a similar enlargement of an absorbent vessel, to whatever class it may belong, can *take up,* and transmit, twice the number of atoms in the same time. On the contrary, any loss of contractile power in the absorbents, (or the extremities of the veins, or imbibing pores, or whatever the nature of the absorbing apparatus may be,) must render their function less active than it is in their natural state. If the preceding view be correct, it follows that the same morbific cause which is calculated to *accelerate* the secreting function, has also a tendency to *reduce* the activity of the function of absorption. As in all cases of local inflammation the dimension of the capillaries is considerably increased, they have a morbid necessity of depositing more than the natural and healthy quantity of materials in the seat of disease; and as, on the other hand, the force of the absorbent vessels is diminished by the same cause, the inevitable consequence is, first, a simple swelling; and, ultimately, an organic change of structure." (pp. 69-70.)

The morbid phenomena in all local diseases are further considered as analogous to those which characterize inflammation. As the properties of the arterial extremities vary in the different tissues, and as the tissues themselves are also innumerable in variety, it is no wonder if their derangement should lead to organic changes so different in character. Such being the state of a part when disease is advancing, the obvious therapeutical indication, after the removal of the morbific cause, is not only to restore the lost balance between the deranged functions of secretion and absorption, but, if necessary, for a time to give the latter a preponderance over the former. This is to be accomplished by local remedies which are capable of *recovering the lost contractility of the minute branches and augmenting the action of the absorbents.* In inflammation, local bleeding, evaporating lotions, poultices, fomentations, liniments, escharotics, and pressure, are all explained as operating in this manner.

Were any one so evil disposed as to attempt to prove that the nature of a disease does not necessarily depend on structure, for the same structure may give rise to several kinds of disease; or to deny that the secerning and absorbent vessels are always in a state of relaxation, the foundation and superstructure built upon it would, unfortunately, be swept away; but this ungracious task we are not disposed to undertake.

The utility of iodine, as an external remedy, is prefaced by some judicious remarks on its therapeutic properties, and on the best mode of applying it in a variety of morbid states. As an internal remedy this substance has now been long administered, but without any knowledge of its action, and often without any well-defined object in view; while its outward curative properties seem to have excited little attention. It is not the object of the present essay to take any notice of its inward administration: it being merely remarked, in passing, that after long trial, it has not been found to cause absorption of the mammæ or testes, as usually represented; that it has failed in removing scrofula, for which it has been said to be a specific; but has been found useful in continued dyspepsia, and some other affections of the mucous membrane of the alimentary canal. In bringing before his medical brethren the merits of a remedy which seems to have been too long neglected, the author, being well aware that, to propose any substance as a cure for numerous complaints, is apt both to occasion distrust in its efficacy, and lay the proposer open to a charge of empiricism, has determined to state nothing which repeated observations have not proved to be correct. He has, likewise, very candidly warned us against its indiscreet use—a caution that it would be well to observe on like occasions.

"In urging a remedy on the attention of the profession, it is necessary, at the same time, to caution the members of that profession against the indiscriminate and indiscreet use of it. Many valuable medicines have fallen into disrepute, and have been altogether discarded from the list, owing to their having been mismanaged or abused. Iodine, though not a *new* remedy, has by no means yet had its effects on the human system fully tested. Properties have been attributed to it of which it is quite devoid, while, on the other hand, it is endued with many therapeutic virtues which it has not been generally known to possess." (p. 81.)

The remedy is to be employed in two forms, but principally in the shape of tincture, made by dissolving two scruples of iodine in an ounce of rectified alcohol, the strength of which may be reduced by the addition of more spirit: the other, an ioduretted solution, consists in dissolving thirty-two grains of iodide of potassium in an ounce of distilled water, and adding to it eight grains of iodine. The latter is not so useful as an external application, as, from its containing the hydriodate, it is rendered acrid, and causes considerable smarting when laid upon an abraded surface, diluted, however, with from seven to ten parts of water, with the addition of a little honey, it forms a good iodine gargle in ulcerated sore-throat. Nevertheless, the alcoholic tincture is preferable in all cases, provided the practitioner can find time to apply it himself—an indispensable condition, apparently, in its use. The strength of the preparation is to be varied according to the *quality* of the skin, and the nature or intensity of the disease. Where the former is thin and delicate, the tincture must be reduced one half by adding spirit; where a part, again, is acutely inflamed, or threatening to slough, it ought at first to be applied undiluted, and afterwards weakened as the necessity for it diminishes. The effect of its application to *healthy* skin is a sensation of heat and smarting; sometimes only a slight and agreeable warmth, followed by desquamation of the cuticle. When too long continued, the skin is liable to form watery vesicles, which give way in a few days to a lotion of spirit and water. When applied to an *inflamed part*, on the contrary, the pain soon becomes deadened, and a sensation of warmth succeeds, but which subsides in a few hours, leaving the patient free from any uneasiness in the seat of disorder. Brought into contact with a sloughing ulcer, a part on the verge of gangrene, or a foul, irritable sore, it is seldom felt by the patient. In a healthy ulcer, a recently-lacerated wound, or any healthy raw surface, it produces generally a very sharp smarting pain for a minute or two, but which ceases and leaves the part in a comfortable state.

The rationale of the operation of this remedy accords, of course, with the author's views respecting the formation and removal of disease; it excites contractility of the capillaries, thereby diminishing secretion, while, at the same time, it increases the activity of the absorbent system. The reader, however, is requested not to take the failure to give a satisfactory reason for its mode of acting, as a justification for rejecting the ascertained *facts* respecting its effects. To prevent repetition, we shall here give the mode of using the iodine, in perhaps one of the worst cases that could come under our notice.

"Suppose we are called to a case of severe inflammation of the leg, in a stout, robust person: the limb is intensely red, hot, swollen, and glossy, all the way from the toes to above the knee; it is double the size of the corresponding one, and so painful as to disturb the general health, as to cause quickness of the pulse, white tongue, thirst, &c. We immediately *paint* the whole limb with the tincture of its full strength, extending its application from the toes to several inches above the upper margin of the inflammation; the remedy is applied with a camel's hair brush. This is all the local application requisite for the present. The limb is directed to be kept in a horizontal posture, and either to be very lightly covered over with a sheet, which must not come in contact with the skin, or else to be left exposed, according to the temperature of the apartment. In less than twenty-four hours, in less than twelve hours, the swelling will be found to have diminished. At the end of twenty-four hours the skin will be seen much corrugated, showing its contents to have become less in bulk, and the circumference of the limb will measure some inches less than the day before. The diminution will be found to have taken place more particularly towards the upper part of the swelling. We now repeat the application of the tincture, of the same strength. In another twenty-four hours the reduction of the swelling will have gone on rapidly, and only a remnant of the disease will be found to exist. The strength of the tincture must now be reduced to one half, and its application continued daily, or less often, according to circumstances, until the limb is well. After the second or third application of the tincture, we sometimes brush the limb over with spirits of wine alone, so as merely to dissolve the iodine which remains on the surface of the skin." (pp. 83-4.)

We will now briefly state the diseases in which iodine forms so useful an external application, premising that the local treatment, however instrumental in reducing the constitutional disturbance, by assisting in the removal of the disease, must not supersede general remedies where these are required. First, then, in *erysipelas*, in whatever situation and of whatever description, the author has found the tincture, applied as above, preferable to leeches, lotions, incisions, scarifications, or caustic. In common *phlegmon*, the same beneficial changes are seen. Where pain and throbbing only exist, it will be found that one application of the full-strength tincture will cut short the disease; and where suppuration has commenced, its repeated use has not only checked the progress of the disease, but caused the deposited matter to be absorbed. In *very* deep-seated inflammation among the muscles, as in the thigh or loins, the efficacy of the remedy is perhaps doubtful. Not a single case of failure was met with, where the tincture was applied in superficial phlegmon before suppuration occurred; and even then the quantity of pus was much less than where poultices were used. In *extensive sloughing of the cellular membrane* after phlegmonous erysipelas in the lower extremities, where the tissue protrudes through ulcerated openings in the skin, and sloughs in large quantities, the tincture is the most valuable application. While the usual remedies have no effect in checking the inflammatory process, the iodine at once arrests it, and gives the living parts a chance of casting off the dead slough. Cases illustrating the utility of the remedy in these three states are adduced, and which are selected from amongst numerous others that could have been brought forward.

In diseases less threatening to life, the remedy is perhaps still more successful. In *acute inflammation of the joints*, it is more efficacious than any local application in common use. Over the knee, if the skin is delicate, it may be applied at first about half its strength, and increased gradually as required. When the hip is affected, the strong tincture should be painted all around the upper part of the thigh and groin. In these cases, the author prefers leeching the joint, and then using the iodine as soon as the bleeding ceases. In *inflammation of the breast*, as soon as the disease is discovered, the tincture of full strength is to be laid extensively over the part, and even if abscess should form, its extent will be limited. In *gout*, its application cuts short the attack; and in anomalous pains of the joints, supposed to be gouty or rheumatic, its effect has been very noted. The tincture is diluted to about two thirds its full strength. In *chronic inflammation and enlargement of the joints*, such as the hip and knee, leeches are first employed, and then the diluted tincture is laid extensively over the part, and repeated every two or three days according to its effects on the skin. The plan is to be persevered in for a period limited only by the duration of the disease. In the ankle or wrist, where the enlargement is of old standing, the iodine lotion is preferable to the tincture, a rag being wetted in it three or four times a day and laid round the joint. The strength of the lotion must be determined by the discretion of the attendant. In *inflammation of the absorbents*, the strong tincture applied along the whole track of vessels, generally suffices to subdue the disease. In *carbuncle*, used before or after incisions, it will dispel the inflammation, and enable the parts to cast off the dead cellular tissue and form granulations. It is equally applicable to *boils* and *buboes*; in the latter cutting short their progress, or else, if used after suppuration, limiting the extent of the abscess. "These," says the author, "are real facts, facts easily tested by trial." In *lupus*, or *noli me tangere*, the strong tincture laid upon the ulcerated surface has cured the disease without any internal remedies; in one case, however, it failed, although assisted by the internal use of the arsenical solution. In *malignant ulcers of the tongue and tonsils*, the tincture of full strength, brushed all over the parts, arrests the affection however threatening. The only internal remedy was the ioduretted solution, in doses of ten drops twice a day in water. In *scrofulous swelling of the glands*, it either resolves the inflammation and causes absorption of the morbid deposits, or limits the formation of matter and assists in the cicatrization of the sore. In *whitlow*, the strong tincture is to be immediately painted over the whole finger or thumb, and repeated in *twelve* hours, unless the morbid sensation has ceased. Where this has been done before suppuration, it never fails to subdue the disease. Should matter exist, a free incision must be made, and the tincture then applied over the finger or hand if swelled. In *chilblains*, the remedy of full strength is to be applied over the sore and beyond the boundary of the surrounding inflammation, and repeated daily for some time. The affected parts should be immersed every night in water as hot as can be borne. When the ulceration looks healthy, and the skin around has lost its livid colour, the strength of the tincture may be reduced. After each application, the sore should be dressed with some stimulating ointment. In cases where the inflammation spreads along the foot or leg, the affected parts must be *painted* with the strong tincture. It is not stated what effect the remedy has before ulceration takes place.

The tincture of iodine has been found preferable to every plan of local treatment in *lacerated, contused, and punctured wounds.* When the accident is one of *simple laceration*, after the blood or dirt are wiped away, every point of its surface is touched over with the remedy, generally of full strength, and its application extended a little distance beyond. After allowing it to dry, the edges are brought together with sticking plaster, which is not renewed for three or four days, when part will be found united and the rest granulating. The latter, with the surrounding skin, is again brushed over, and then dressed with common wax-ointment. The cure is generally rapid. Where *contusion* only exists, the tincture is applied every day or two to the surface, and quickly causes absorption of the extravasated blood. Where there is a *combination* of laceration and contusion, the treatment is compound. The surface of the wound and contusion is brushed over, and the edges of the former then approximated and kept together by plaster or a roller. The remedy is reapplied according to the necessity of the case. In *punctured wounds*, from whatever cause, the remedy liberally applied is used with that undeviating success which it exerts in local diseases and injuries attended by inflammation. In such cases it should be allowed to insinuate itself freely into the wound, and be thickly painted upon the surrounding skin. In *burns* and *scalds* it seems to act as it does in erysipelas. When the integuments are not destroyed, although the cuticle may be in blisters, one or two applications of the tincture, of moderate strength, subdue the pain and redness, after which the parts only require to be kept free from injury. Further trials will be necessary to determine its effects where the violence has been extensive. Lastly, the remedy is eminently successful in *ulcers*. We have already noticed its efficacy in lupus. Several cases of chancre have yielded sooner than to the ordinary treatment, while in the malignant ulcerations about the lips, tongue, or tonsils, no topical remedy we possess is equal to it. In all cases of irritable, or sloughing sores, the tincture of full strength should be applied to the surface and surrounding skin. After being allowed to remain some time, the ulcer should be covered with simple ointment in preference to a poultice. The application is repeated daily till the sore becomes clean and healthy, when the granulations may be touched with the diluted tincture every two or three days. Under this plan, the cavity of the ulcer fills up rapidly. Besides these various affections, the tincture has been employed with good effect in gouty and rheumatic swellings of the small joints from thickening of their ligaments, fistulous openings, malignant warts or adventitious excrescences, ganglions, the stinging of wasps, diseases of the spine, ununited fractures, hernia humoralis, inflamed urethra and chordee, inflammation of the bursæ, chronic ophthalmia, and opacities of the cornea (much diluted), dissection wounds, or scratches exposed to the dead body in dissection, &c. The strength of the remedy in the several cases must depend upon the judgment of the practitioner.

We have been anxious to present our readers with a short sketch of the various cases in which iodine has been found so efficacious as an external remedy. We have done so, with the twofold view of inducing them to study the work itself, and put the remedy, as soon as possible, to the test of experience. The statements respecting it are put forth in a plain, lucid, and straightforward manner, and without any desire to exaggerate its effects. The trials to which it has been subjected have been in every instance numerous, and apparently run over a period of ten years. We do not pause, therefore, to make any comments on the treatment here recommended; the facts to us, and we presume to many others, are novel and extraordinary; and it would be unbecoming to say anything of a line of practice which we have never witnessed, far less followed out. We trust that those who enjoy the opportunities will give it a fair and impartial trial, and communicate the results in such a way that they will be diffused amongst those whose means of acquiring information are more limited. Its efficacy appears so surprising, that this will only be an act of public justice towards the author and mankind; and we doubt not that the former will agree with us in courting every enquiry; if, as we expect, it shall be found considerably less *heroic* in other hands—a circumstance readily explicable without the least imputation on the author's truth and judgment—Mr. Davies will still merit the thanks of his professional brethren; and we strongly recommend his Essay on Iodine to their perusal.

